# Laryngeal Tuberculosis in Pregnant Women: A Case Report and Review of the Literature

**DOI:** 10.7759/cureus.3545

**Published:** 2018-11-05

**Authors:** Jorge Luis Hurtado Alegre, Anita Trigoso Gutierrez, Eduardo Matos Prado, Jorge Huaringa Marcelo

**Affiliations:** 1 Infectious Diseases, Hospital Nacional Arzobispo Loayza, Universidad Nacional Mayor De San Marcos, Lima, PER; 2 Internal Medicine, Hospital Nacional Arzobispo Loayza, Universidad Nacional Mayor De San Marcos, Lima, PER

**Keywords:** laryngeal tuberculosis, dysphonia, pregnant, laryngoscopy

## Abstract

Tuberculosis is the most frequent granulomatous disease but the involvement of the larynx is rare. Immunosuppressed patients have a higher risk of developing this clinical form due to primoinfection or reactivation of latent tuberculosis. It is common to confuse the diagnosis of laryngeal tuberculosis with laryngeal cancer because they have similar macroscopic lesions and both produce dysphonia of chronic evolution. We present the case of a pregnant woman with chronic dysphonia, dysphagia, and odynophagia, diagnosed initially with laryngeal cancer after the first laryngoscopy. However, the patient also presented with fever, productive cough, weight loss, and dyspnea. The sputum sample showed a positive result for acid-fast bacilli (AFB) test; chest X-ray was showed bibasal nodular lesions with a predominance of right hemithorax and reticular opacities in left apex. A new laryngoscopy revealed a mamelonated tumor in the arytenoid cartilage, which led to the initiation of the antituberculous treatment without confirming the diagnosis by biopsy, with clinical improvement and no serious sequelae at the end of treatment.

The reason for presenting this case is to consider the possibility of tuberculosis in areas of high endemicity, in patients who have a laryngeal tumor of probable neoplastic etiology, and that a biopsy is not necessary for the diagnosis of laryngeal tuberculosis in cases associated with pulmonary symptomatology.

## Introduction

Laryngeal tuberculosis represents less than 1% of all the cases of tuberculosis and most of them are associated with pulmonary involvement. The incidence is higher in pregnant women due to their disturbed cellular immune system, where the inflammatory activity of Th1 cells is reduced, resulting in decreased production of interferon-gamma. This leads to a suppressed cellular immune response [1-2] and increases the risk two times more compared to that in a non-pregnant woman, besides the disease evolution is usually faster and more aggressive [3-5].

The clinical presentation of laryngeal tuberculosis is similar to laryngeal cancer, as progressive dysphonia is a characteristic symptom in both cases [6-7]. Laryngeal cancer is approximately 40 times more frequent than laryngeal tuberculosis, which explains why in some series of cases of laryngeal tuberculosis, the most important differential diagnosis is laryngeal cancer [8-9]. Even laryngoscopy can show similar lesions between both pathologies [9].

Laryngeal tuberculosis can be divided into primary (without pulmonary involvement) or secondary (with pulmonary involvement). Generally, the patients show a positive response towards the specific treatment with antituberculosis drugs, but it’s necessary to start it as soon as possible in order to avoid the sequelae [10]. 

## Case presentation

We present the case of a 28-year-old pregnant woman with 35 weeks of gestation, from Loreto, Peru. She had four months of progressive dysphonia, loss of weight (5 kg), dry cough, and dysphagia which at the start was only for solid food and then even for liquids. One month before admission, the patient presented with odynophagia, fever, night sweats, and a productive cough. She was admitted to the emergency room due to respiratory distress and hemoptysis. At the time of her admission, she brought a laryngoscopy report that concluded laryngeal cancer; although a biopsy was not performed.

During the physical examination, she was in a bad general condition. Tachypnea, dysphonia, and a decrease of the subcutaneous cellular tissue were evident. The cardiac and respiratory frequency were increased (110 bpm and 14 vpm) and a temperature of 39°C was recorded. No other alterations were found in the rest of the examination.

**Figure 1:**
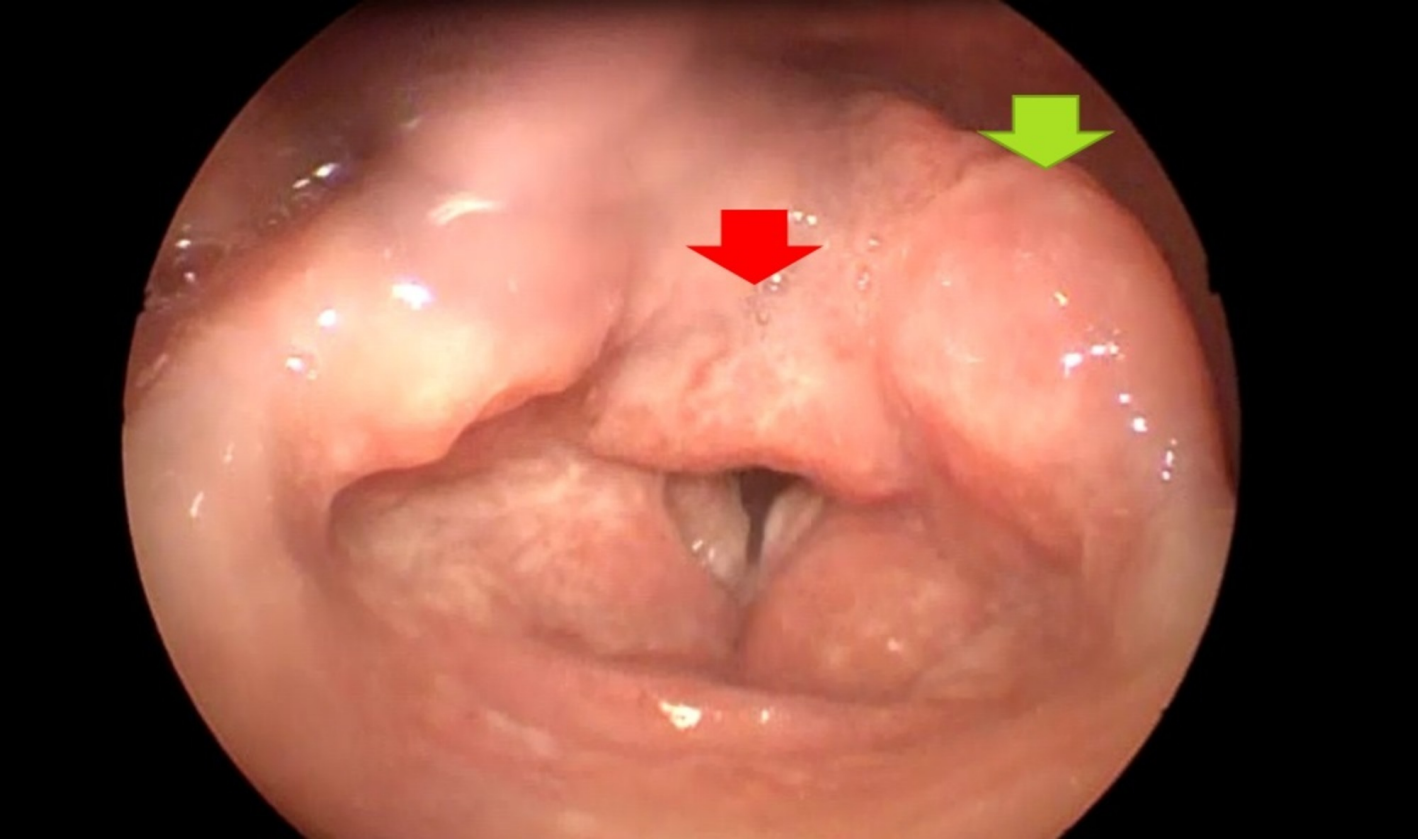
Mamelonated laryngeal tumoration that compromised the arytenoid cartilage (green arrow) and the interarytenoid notch (red arrow)

**Figure 2:**
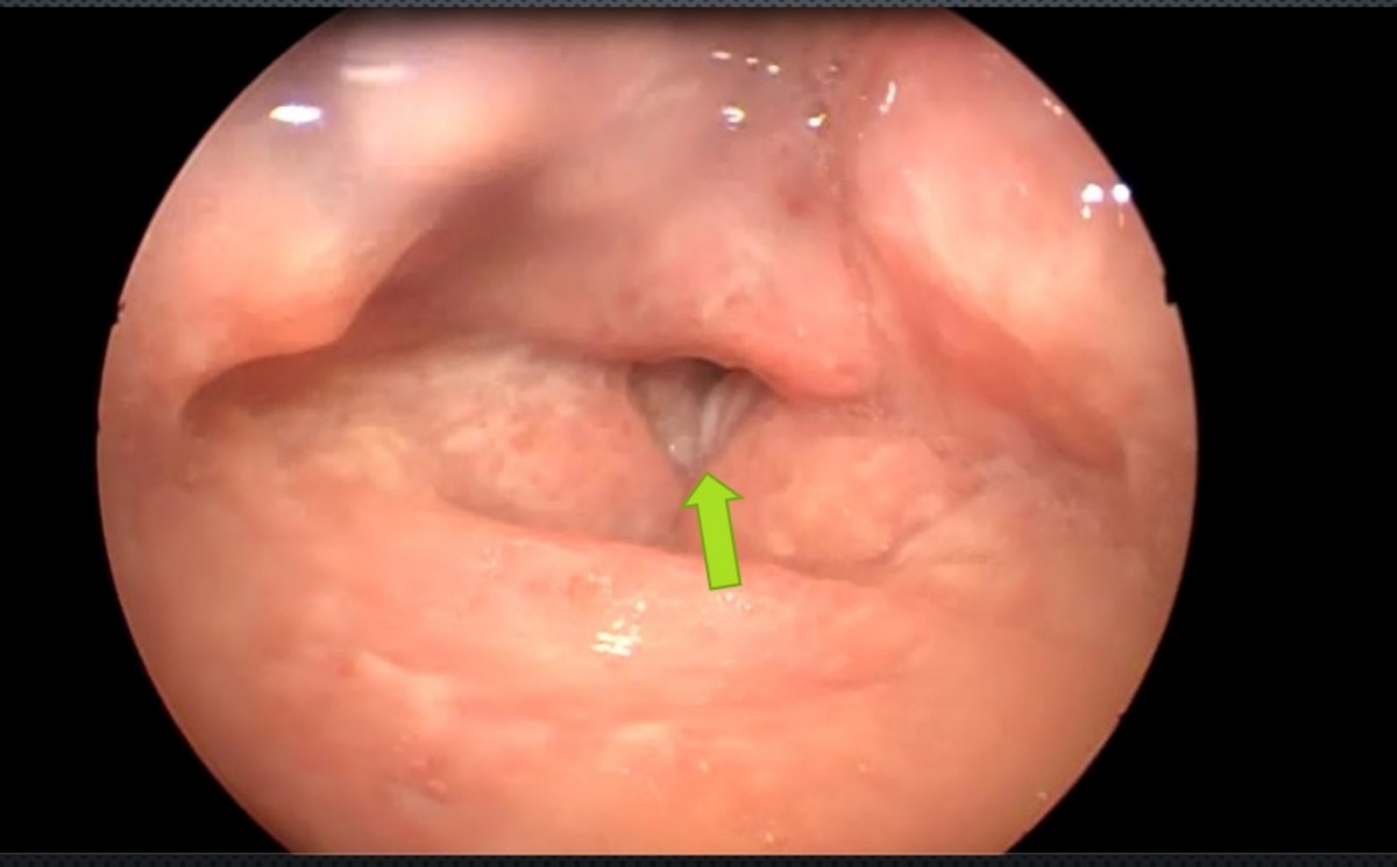
Vocal cords presented irregularities with predomination of the right side (green arrow) and their limited mobility

Due to the history of chronic dysphonia and the laryngoscopy report that indicated the existence of laryngeal compromise, a differential diagnoses were proposed: laryngeal cancer and laryngeal tuberculosis. The diagnosis revealed respiratory and systemic symptoms suggesting the infectious etiology as the cause. A new laryngoscopy was performed, which reported a mamelonated laryngeal tumoration that compromised the arytenoid cartilage and the interarytenoid notch; the vocal cords presented irregularities with predomination of the right side, and their mobility was limited (Figures 1-2) (Video 1). 

**Video 1:** Laryngoscopy features

**Figure 3:**
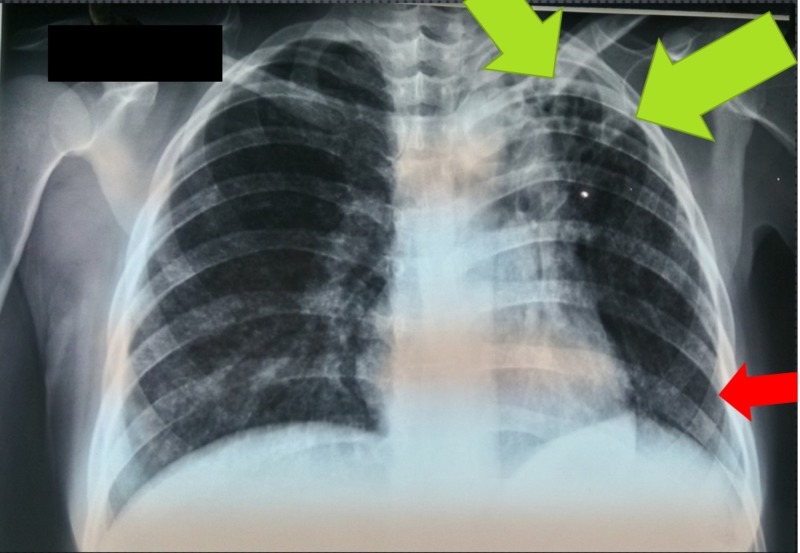
Bibasal lesions of fine nodular pattern with a predominance of the right hemithorax, reticular opacities at the left apical level (green arrows) and an ipsilateral elevation of the hemidiaphragm (red arrow)

Other tests were requested to evaluate the active tuberculosis disease at pulmonary stage; a sputum bacilloscopy showed positive result (+/+++) and the chest radiography showed bibasal lesions of fine nodular pattern with predominance of the right hemithorax, reticular opacities at the left apical level and an ipsilateral elevation of the hemidiaphragm; as described earlier, it was raised with a high suspicion that the case could be laryngeal tuberculosis secondary to lung infection as the starting point (Figure 3). 

Nevertheless, it was not possible to perform a biopsy of the laryngeal lesions due to the level of damage that was observed in the larynx and that could be caused during the procedure; besides, the infectious etiology was already clear due to the positive sputum smear result. Empirical treatment with drugs (isoniazid, rifampicin, pyrazinamide, ethambutol) for sensitive tuberculosis was started, and after a week, the patient presented significant clinical improvement (resolution of fever, dyspnea, cough, and dysphonia), which confirmed the presumptive diagnosis and she was discharged from the hospital after being adviced to continue treatment with an ambulatory control.

The following sputum smears were negative from the second month, until after the treatment, so it was considered as healed. No adverse reactions to the drugs or serious sequels of the disease were reported.

## Discussion

Tuberculosis is the leading cause of death from a single infectious disease that affects, more frequently, the regions with low economic resources. In countries such as Peru, the burden of disease is high [11]. However, laryngeal tuberculosis is a rare presentation of tuberculosis, since it represents less than the 1% of all cases of tuberculosis [3-5]. The incidence of this disease is increasing due to high frequency of conditions that lead to the deterioration of the function of the immune system and that are risk factors for this disease: human immunodeficiency (HIV) virus infection, advanced age, diseases or immunosuppressive treatments [10]. 

Pregnancy is an immunosuppressive condition and represents a risk factor for primary infection or reactivation of latent tuberculosis [3-4]. During pregnancy, the progression of the disease is faster and with higher mortality rates [3]. The available data suggest that in high-burden countries, the prevalence of active tuberculosis among pregnant women ranging from 0.07% to 0.5% were found among HIV-negative women, and between 0.7% and 11% among HIV-positive women. However, the epidemiological information on laryngeal tuberculosis in pregnancy is scarce, since few countries perform systematic tests for the detection of tuberculosis in pregnant women [1,3,12]. 

Laryngeal tuberculosis usually occurs through bronchogenic, hematogenous or lymphatic dissemination from the lung and, in rare cases, the laryngeal lesion is primary; which means, there are no lesions at the pulmonary level and the probable infection route is by direct dissemination of the bacilli by inhalation [10]; in the present case, the infectious route was probably bronchogenic, because the chest radiography revealed compatible alterations with pulmonary tuberculosis.

The Center for Disease Control (CDC) classifies laryngeal tuberculosis as a highly infectious form of tuberculosis [13]; nonetheless, this is attributed to the extent and severity of the pulmonary lesions [14]. In the primary location, the lesions are small, the main symptom is dysphonia and they are not associated with an increase in the cough frequency, so it does not get a sufficient number and size of aerial particles (droplets of Flügge), which are responsible for the transmission [15]. 

Tuberculosis is the most frequent granulomatous disease of the larynx [4] and generally can affect more than one of these areas: the true vocal cords, epiglottis, false vocal cords, arytenoids, posterior commissure, and subglottic area. On performing laryngoscopy in patients with laryngeal tuberculosis, the types of lesions observed were: granulomatous, ulcerative, polypoid and nonspecific, the first one being the more frequent one [7,16,17]. Scarring and fibrosis areas are produced at the subepithelial space that alter the movement of the vocal cords to close and open the glottis in the vibratory cycle of phonation that clinically manifests as progressive evolution dysphonia [18]. 

The signs and symptoms of laryngeal tuberculosis in order of frequency, according to reviewed series of cases, are dysphonia, weight loss, dysphagia, and odynophagia [4,5,16]. Laryngeal carcinoma can present as main symptom dysphonia, similar to laryngeal tuberculosis, nevertheless, some reviews found that there are other symptoms that are more suggestive of tuberculosis (fever, weight loss, and cough), besides odynophagia which is not common in cases of laryngeal cancer; however, there is still no evidence of the reason this symptom is found more frequently in tuberculosis [19]. 

The differential diagnosis is broad and includes neoplasms, infectious and autoimmune diseases; but laryngeal neoplasia is the most similar in clinical and laryngoscopic findings [4,6,8,19]. Case reports of laryngeal tuberculosis were reviewed in which up to 80% of the initial diagnosis was neoplasia, which is explained by the low frequency of this disease and that symptoms can simulate other more common pathologies (laryngeal cancer is at least 40 times more frequent than laryngeal tuberculosis) [8]. Consequently, the diagnosis can be wrong or overlooked, causing a delay in the start of treatment and increased mortality due to complications, increased transmission between close contacts, and increasing the risk of sequels.

**Figure 4:**
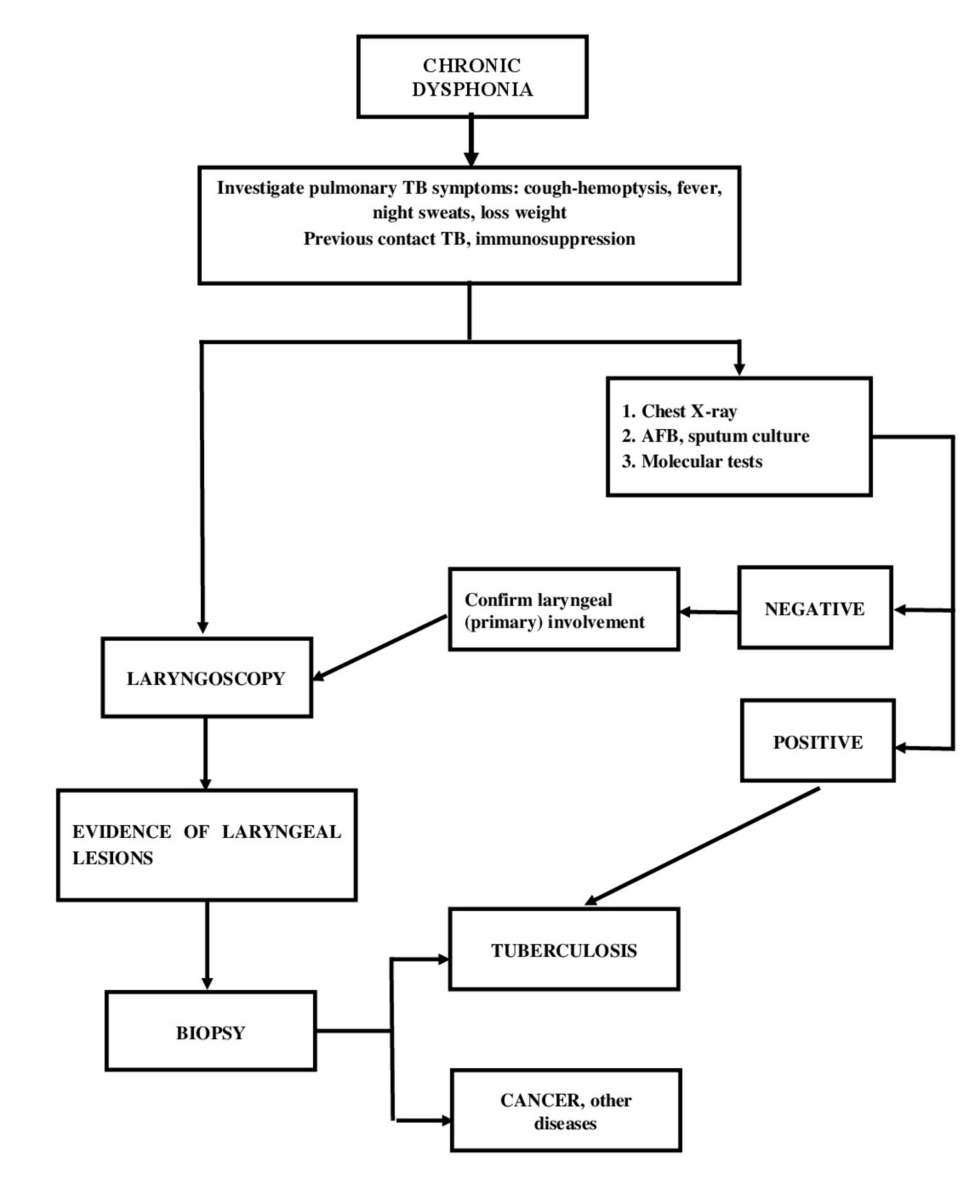
The diagnostic algorithm of chronic dysphonia proposed by the authors

Based on the reviewed literature, a diagnostic algorithm is proposed for a patient with chronic dysphonia from an area of high tuberculosis endemicity (Figure 4). Therefore, a thorough history should be taken of a patient with chronic dysphonia in order to identify risk factors, history of contact and other associated symptoms such as fever, night sweats, weight loss, productive cough, hemoptysis and odynophagia (which occurred in this case). Then, auxiliary examinations should be requested such as chest X-ray, cultures, other available molecular tests, to confirm the pulmonary involvement as the primary focus. However, if the auxiliary exams for pulmonary tuberculosis are negative, a laryngoscopy should be performed with a biopsy to confirm a possible primary laryngeal tuberculosis or rule out any other pathology.

Once the diagnosis is confirmed, the treatment is based on antituberculous drugs administered according to the sensitivity results for a minimum of six months. The cure is confirmed through sputum microbiological controls, chest X-ray, and laryngoscopy. The favorable response to treatment and the positive results of the auxiliary tests for tuberculosis confirmed the diagnosis of laryngeal tuberculosis, in this case, even without the result of the biopsy. The treatment has shown to achieve regression of laryngeal, pulmonary, and systemic symptoms [9,16,20]. 

Regarding the possible sequelae because of the laryngeal commitment of the disease, the principal is dysphonia which results from the subglottic stenosis, muscular affection, and vocal cords paralysis when the cricoarytenoid joint or recurrent laryngeal nerve is invaded [10]. 

An important aspect in the management of the case was that even without the laryngeal biopsy result, the diagnosis could have been established, such as in our case. Some authors mention that it could be dispensed in cases with clinical and microbiological studies consistent with tuberculosis; it also avoids causing greater damage when performing the biopsy [9]. 

## Conclusions

From the case, we can conclude that the result of a biopsy is not necessary for the diagnosis of laryngeal tuberculosis in patients with pulmonary or systemic symptoms of active tuberculosis (secondary laryngeal tuberculosis). However, the biopsy will indicate the diagnosis of primary laryngeal tuberculosis (without pulmonary lesions) or help rule out any other pathology.

## References

[1] Bates M, Ahmed Y, Kapata N, Maeurer M, Mwaba P, Zumla A (2015). Perspectives on tuberculosis in pregnancy. Int J Infect Dis.

[2] Sulis G, Pai M (2018). Tuberculosis in pregnancy: a treacherous yet neglected issue. J Obstet Gynaecol Can.

[3] Geier J, Orlando B (2018). Pulmonary and laryngeal tuberculosis in a 25-weeks' gestation parturient, diagnosed after failed tracheal intubation. Int J Obstet Anesth.

[4] Benwill JL, Sarria JC (2014). Laryngeal tuberculosis in the United States of America: a forgotten disease. Scand J Infect Dis.

[5] Kurokawa M, Nibu KI, Ichimura KI, Nishino H (2015). Laryngeal tuberculosis: a report of 17 cases. Auris Nasus Larynx.

[6] Suhail A, Ahmed MS, Sobani ZU, Ghaffar S (2018). Laryngeal tuberculosis presenting as laryngeal carcinoma. J Pak Med Assoc.

[7] Lucena MM, da Silva FD, da Costa AD (2015). Evaluation of voice disorders in patients with active laryngeal tuberculosis. PLoS One.

[8] Wang CC, Lin CC, Wang CP, Liu SA, Jiang RS (2007). Laryngeal tuberculosis: a review of 26 cases. Otolaryngol Head Neck Surg.

[9] Ling L, Zhou SH, Wang SQ (2010). Changing trends in the clinical features of laryngeal tuberculosis: a report of 19 cases. Int J Infect Dis.

[10] Lim JY, Kim KM, Choi EC, Kim YH, Kim HS, Choi HS (2006). Current clinical propensity of laryngeal tuberculosis: review of 60 cases. Eur Arch Otorhinolaryngol.

[11] (2018). Global tuberculosis report. World Health Organization.

[12] Sugarman J, Colvin C, Moran AC, Oxlade O (2014). Tuberculosis in pregnancy: an estimate of the global burden of disease. Lancet Glob Health.

[13] (2018). Guidelines for the investigation of contacts of persons with infectious tuberculosis. Recommendations from the National Tuberculosis Controllers Association and CDC. Morb Mortal Wkly Rep.

[14] Pio A (2018). The infectiousness of laryngeal tuberculosis. Int J Tuberc Lung Dis.

[15] Rieder HL (2018). The infectiousness of laryngeal tuberculosis: appropriate public health action based on false premises. Int J Tuberc Lung Dis.

[16] Zhao N, Zhang Y, Li K (2017). Rigid laryngoscope manifestations of 61 cases of modern laryngeal tuberculosis. Exp Ther Med.

[17] Paulauskienė I, Mickevičienė V (2016). Dysphonia-the single symptom of rifampicin resistant laryngeal tuberculosis. Open Med (Wars).

[18] Özüdogru E, Cakli H, Altuntas EE, Gürbüz MK (2018). Effects of laryngeal tuberculosis on vocal fold functions: case report. Acta Otorhinolaryngol Ital.

[19] Smulders YE, De Bondt BJ, Lacko M, Hodge JA, Kross KW (2009). Laryngeal tuberculosis presenting as a supraglottic carcinoma: a case report and review of the literature. J Med Case Rep.

[20] Lodha JV, Sharma A, Virmani N, Bihani A, Dabholkar JP (2015). Secondary laryngeal tuberculosis revisited. Lung India.

